# Immature and mature myocardium in the pathophysiology of hypertrophic cardiomyopathy^☆^

**DOI:** 10.1016/j.jmccpl.2026.100859

**Published:** 2026-07-31

**Authors:** Jan M. Federspiel, Steven Lee Medarev, Jochen Pfeifer, Vivek P. Jani, José Renato Pinto, Maicon Landim-Vieira

**Affiliations:** a Institute of Legal Medicine, Saarland University, Faculty of Medicine, Homburg, Saar, Germany; b Department of Biomedical Sciences, Florida State University College of Medicine, Tallahassee, Florida, USA; c Department of Pediatric Cardiology, Saarland University Hospital and Faculty of Medicine, Homburg, Saarland, Germany; d Division of Cardiology, School of Medicine, Department of Biomedical Engineering, Johns Hopkins University, Baltimore, MD, USA; e Department of Biology, Illinois Institute of Technology, Chicago, IL, USA

**Keywords:** Hypertrophic cardiomyopathy, Hypertrophic obstructive cardiomyopathy, Myocardial adaptation, Embryology, Cardiac remodeling

## Abstract

Hypertrophic cardiomyopathy (HCM) is the most common inherited cardiomyopathy. The pathognomonic finding is the thickening of the myocardial layer, usually of the left ventricular wall. This hypertrophy is often caused by variants in genes encoding sarcomeric proteins. Additionally, non-genetic factors contribute to the complex pathophysiology. Interestingly, these genetic and non-genetic factors result in quite uniform pathological changes, e.g., diastolic dysfunction and hypercontractility can be regarded as pathophysiological hallmarks of the disease. A potential explanation for this might be that embryological factors along with a first adaptive and later maladaptive myocardial remodeling prime myocardial hypertrophy. The present review compares the immature and mature myocardium to further assess the impact of embryological factors on the pathophysiology of HCM. According to the literature, there are differences between the immature and mature myocardium affected by sarcomeric variants at the molecular level. At the same time, the early postnatal period seems to be fundamental to sarcomeric alterations that later drive hypertrophy. Given the shared down-stream pathological changes, fetal-like reprogramming might be the pathophysiological molecular merging point of immature and mature myocardium finally driving the maladaptive hypertrophic remodeling in HCM. More research on such embryonic factors might allow for further HCM-specific targeted therapy.

## Graphical abstract

In hypertrophic cardiomyopathy (HCM) pathophysiology, common downstream alterations are observed, including myocardial thickening, energetic disturbances, and hypercontractility. This narrative review compares the immature myocardium (blue areas) and the mature myocardium (yellow areas). During the early postnatal period, fundamental sarcomeric alterations emerge and later drive hypertrophic remodeling. Fetal-like reprogramming may represent a shared downstream converging point in the pathophysiology of both immature and mature myocardium (green areas). Further research on such embryonic factors might allow for further HCM-specific targeted therapy.***Abbreviations:*** HCM – hypertrophic cardiomyopathy. (Created in BioRender. Federspiel, J. M. (2026) https://BioRender.com/dch4tmp, agreement number *ET29J28BMV*.)

**Figure:**
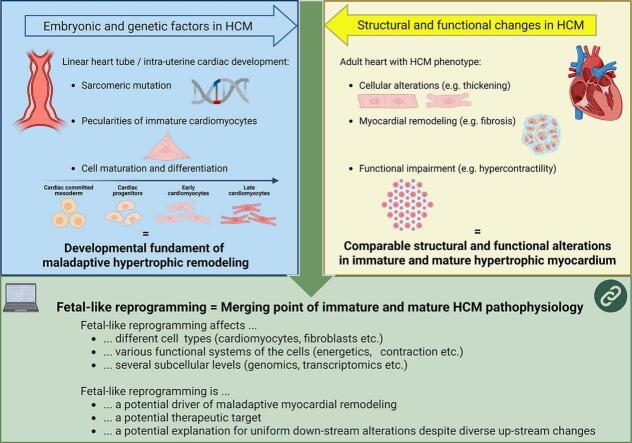

## Abbreviations

**Table:** 

(α−/β-)MHC	(alpha/beta)-myosin heavy chain
ATP	adenosine triphosphate
CAMKII	Ca^2+^/calmodulin-dependent protein kinase II
DRX	disordered-relaxed state
EGR1	early growth response 1
ERK	extracellular signal-regulated kinase
HAND2	heart- and neuralcrest derivatives expressed protein 2
HCM	hypertrophic cardiomyopathy
iPSC	induced pluripotent stem cells
LV	left ventricle
MAPK	mitogen-activated protein kinase
MEF2	myocyte-specific enhancer factor 2
MyBPC3	myosin binding protein C3
MYH6/7	myosin heavy chain 6/7
NFAT	nuclear factor of activated T-cells
PCr	phosphocreatine
PKA	protein kinase A
RNA	ribonucleic acid
ROS	reactive oxygen species
SERCA	sarco/endoplasmatic reticulum calcium adensine triphospatse
SRX	super-relaxed state
TNNT	troponin t

## 1 Introduction

Hypertrophic cardiomyopathy (HCM) is considered the most common inherited cardiomyopathy [1]. It is estimated to affect approximately 1 in 500 young adults in the United States [2]. The pathognomonic finding in HCM is the thickening of the myocardial layer, often of the left ventricle (LV) including the interventricular septum [3], [4]. However, the right ventricle is also frequently involved. For example, a necropsy study reported hypertrophy of the right ventricle in 64% of 72 hearts from individuals diagnosed with HCM [5]. With HCM representing a global health burden [6], understanding its pathology and pathophysiology advances treatment strategies, including pharmacological and (e.g., myosin-inhibitors [7], [8], [9]), invasive approaches (e.g., surgical myectomy [10], [11] or alcohol septal ablation [11], [12]), and diagnostic tools (e.g., echocardiography [13] or magnetic resonance imaging [14]). Importantly, embryological factors in HCM were recognized many years ago, for example by Bulkley and colleagues as early as 1977 [15]. These developmental factors may represent a foundation of disease pathophysiology, supporting the concept of a developmental origin of HCM [16]. In this context, cardiac development may help explain important features of HCM, such as the presence of subclinical disease [17]. A recent review further proposed that embryonic factors may underlie the predisposition of the interventricular septum to pathophysiological hypertrophic remodeling [18].

The formation of the human heart begins in the second week of pregnancy [19] and ultimately results in two atria separated by the interatrial septum, two ventricles separated by the interventricular septum, and four heart valves that direct the blood flow. Together with the inflow and outflow of the great vessels, the heart drives the two circulatory systems arranged in series: the low-pressure pulmonary circulation and the high-pressure systemic circulation [20]. This process of cardiac formation is complex from every perspective, including understanding how the three-dimensional architecture is formed [21], how the heart begins to beat [22], and what occurs at the molecular level in general [23] or at the genetic level in particular [24]. A synopsis of these different "levels" of cardiac development is therefore crucial for understanding this process. For example, the polarity of the heart tube is closely linked to molecular and genetic processes [20]. Understanding cardiac development not only expands knowledge on congenital heart disease [25] and improves the clinician's understanding of pathophysiology [26], but also supports new advancements in the field of cardiovascular sciences more broadly. For instance, reactivation of the so-called "fetal gene program" has been shown to be important in adult heart disease [27], [28], [29], [30]. This fetal-like reprogramming is regarded as potential therapeutic target in failing hearts in general [29] and in hearts exposed to increased afterload [31]. Thus, the long-standing assumption that the heart is a post-mitotic organ has become obsolete [19].

To further explore the importance of embryonic factors and aspects of cardiac development in HCM, this narrative review: (I) summarizes the pathology and pathophysiology of the mature diseased myocardium, (II) compares mature and immature hypertrophic myocardium, and (III) outlines the reactivation of fetal-like gene programs in diseased mature myocardium to support the importance of developmental mechanisms in hypertrophic myocardium regardless of age or developmental stage.

## 2 Hallmarks of mature hypertrophic myocardium

### 2.1 General and genetic aspects

The genetic basis for HCM – a sarcomeric disease – mainly involves variants in sarcomere proteins. Several variants have been identified in thick filament proteins, including myosin-binding protein C (MyBPC3) and myosin heavy chain (MHC), as well as in thin filament proteins (e.g., the troponin complex [32], [33], [34], tropomyosin, and actin), with thick filament variants encompassing the majority [2], [35]. However, it is estimated that ~50% of patients are “sarcomere-negative” (i.e., without a variant in the respective sarcomeric genes) [36] which is likely due to undetected genetic variants, variants in other structural proteins within the cardiomyocyte, or environmental causes [37], [38]. Different variants, however, are associated with distinct clinical presentations and findings. For example, thick filament variants are associated with more severe LV obstruction, whereas thin filament ones are more often associated with systolic dysfunction, restrictive LV filling, and less prevalent obstruction [39], [40]. Interestingly, patients carrying HCM-linked thin filament variants are more likely to progress to symptomatic heart failure [39].

Detailed analyses on a cellular and subcellular level can help to understand and characterize the actual pathological changes in a given variant, for example type 2 ryanodine receptor (*RYR2*) [41] or troponin T2 (*TNNT2*) [42]. This approach can help establish the actual pathological and clinical relevance of a (new) variant. For such a clinical purpose, human iPSC-derived cardiomyocytes may be particularly suitable, as they can be used to model different clinical presentations of HCM [43] or to develop or test new therapeutic strategies [44].

The typical hallmarks of HCM observed in mature ventricular myocardium include asymmetric LV hypertrophy, LV outflow tract obstruction in cases of hypertrophy of the interventricular septum, reduction in LV cavity size, hypercontractility, and diastolic dysfunction during isovolumic relaxation and filling [3], [4], [45]. From a clinical perspective, the presence or absence of LV outflow tract obstruction (i.e., obstructive HCM) has a major impact on patients' symptoms and treatment. For example, patients diagnosed with severe LV outflow tract obstruction can benefit from a septal reduction therapy (such as surgical myectomy) or myosin inhibition therapy [2], [7], [8], [46]. LV outflow tract obstruction is often related to systolic anterior motion of the mitral valve, resulting in mitral regurgitation, mainly due to the Venturi effect [47], [48] and anatomical alterations of the mitral valve apparatus [49]. Correlations between the presence of obstruction requiring intervention and the presence of thick versus thin filament variants are lacking. Interestingly, sarcomere-negative patients often exhibit severe LV outflow tract obstruction [38], [50].

### 2.2 Mechano-energetic uncoupling and hypercontractility

In general, studies in myocardium from human patients and animal models of HCM have shown disturbances in the biochemical states of myosin [51], altered Ca^2+^ homeostasis [52], metabolic dysfunction [53], and so-called mechano-energetic uncoupling, for example reflected by a reduced phosphocreatine (PCr)-to-adenosine triphosphate (ATP) ratio [54]. Such molecular alterations are substrates for pathological myocardial thickening and arrhythmogenicity, initiating a vicious cycle of maladaptive remodeling [55]. While some aspects of the molecular mechanism by which sarcomere protein mutations elicit dysfunction are similar across different genetic variants, several differ despite the overtly similar clinical phenotype. For example, myocardium carrying myosin heavy chain 7 (*MYH7*) variants [56] along with some *TNNT2* variants [32], [40], [56], [57] typically show unaltered or reduced maximum Ca^2+^-activated tension. Calcium-activated tension, however, is largely preserved in myosin binding protein C variants [58], though in most studies the variance is quite high, suggesting another potential confounding factor. What they have in common is increased Ca^2+^ sensitivity, albeit to varying degrees [32], [59], [60]. This is largely thought to be the mechanism of hypercontractility in HCM, as at physiologic Ca^2+^ concentrations, the generated tension is significantly higher than in the control preparations [61].

While it is true that many variants on their own induce changes in Ca^2+^ sensitization, the role of phosphorylation of cardiac troponin I by protein kinase A (PKA) should not be underestimated [62], [63], [64]. Under physiological conditions, β1-adrenergic stimulation leads to an increased heart rate (i.e., a chronotropic effect), increased contractility (i.e., an inotropic effect), and faster relaxation (i.e., a lusitropic effect), mainly mediated by PKA activation [62]. As part of normal adrenergic stimulation, PKA phosphorylates cardiac troponin I at serine 23 and serine 24, which induces Ca^2+^ desensitization. Despite this Ca^2+^ desensitization, overall contractility increases because tension in the diastolic Ca^2+^ range is reduced, allowing for greater filling during diastole and, by the Frank-Starling mechanism, increased contractility. Additionally, studies in mouse models suggest that phosphorylation of serine 23 and serine 24 may prevent myocardial hypertrophy [63]. Many HCM variants have been found either to interfere with access to serine 23 and serine 24, resulting in reduced phosphorylation, or to alter cardiac troponin I function so that the sarcomere is not desensitized to Ca^2+^ despite serine 23 and serine 24 phosphorylation [59]. Furthermore, analyses in rodent models up to 15 months of age showed that long-term ablation of PKA-mediated phosphorylation contributes to impaired ventricular filling [64]. These analyses also showed that long-term PKA ablation aggravates the excitation-contraction uncoupling seen in HCM and is therefore associated with a metabolic burden [64]. In patients diagnosed with HCM who developed heart failure, downregulation of the β-adrenergic system results in overall reduced cardiac troponin I phosphorylation at serine 23 and serine 24 [59].

Other mechanisms for hypercontractility in HCM include increased ATP-utilization (i.e., increased tension cost), accelerated actin-myosin cross-bridge kinetics, and an increase in the number of myosin heads available for force generation [65], [66]. Increased ATP-utilization can result from either an acceleration of the myosin ATPase rate or an increase in the proportion of high-energy utilizing myosin heads. This increase in demand yields a net energy deficit, resulting in perturbations to high energy phosphates (i.e., reduction in PCr/ATP ratio) and contribute to mitochondrial dysfunction, first observed in isolated myofibrillar preparations isolated from symptomatic patients with R403Q *MYH7* variant [67], [68]. Moreover, ATP deficits correlate with left atrial dilatation in HCM patients, further supporting energetic perturbations as critical to disease pathogenesis [69].

Cross-bridge kinetic rates describe the rates of myosin head attachment and detachment to thin filaments. Acceleration in myosin attachment or deceleration in detachment results in an increase in the duty ratio, resulting in hypercontractility in HCM [67]. Duty ratio (i.e., time in which myosin is bound to actin) is defined as *f*/(*f* + *g*), where *f* is the apparent rate of myosin attachment to force-generating states and *g* is the apparent rate of detachment from those states. Patients carrying *MYBPC3* truncating variants exhibit haploinsufficiency, and mouse models of truncating *MYBPC3* variants show accelerated kinetics [70]. In mice, however, this results in profound systolic dysfunction with an inability to maintain systolic ejection, which is not observed in human HCM [70]. Studies on tissue from such patients may show accelerated kinetics [69], [71], though the variance is high, and many have reported no statistically significant changes [72]. Studies on *MYBPC3* missense variants, however, have identified few changes in cross-bridge kinetics, supporting the idea that the role of MYBPC3 is more complicated than simply actin-myosin cross-bridge kinetics [72], [73]. Regardless of the role it plays, it appears that disturbances in MYBPC3 function contribute to the pathophysiology of HCM. Furthermore, reduced ATP alone can slow the rate of myosin detachment, thereby integrating the two mechanisms [69].

## 3 Comparison between immature and mature hypertrophic myocardium

Maturation causes differences between immature and mature myocardium, as it represents a major physiological transition involving changes in myocardial metabolism [74] and adaptation to altered loading conditions. For example, recurrent stretching of cardiomyocytes appears to induce both maturation and differentiation during development [75], [76]. However, pathophysiological changes can also be observed in maturing cardiomyocytes [75]. From a pathophysiological perspective, the distinction between immature and mature myocardium appears to be somewhat fluid. Mature myocardium may undergo fetal-like reprogramming under pathophysiological conditions [27], [28], [29], [30] (see below). Perhaps because of this indistinct molecular separation between mature and immature myocardium, studies addressing the molecular mechanisms of hypertrophy in immature myocardium are, to the author's knowledge, comparatively rare. Nevertheless, despite maturation-related differences, there also appears to be similarities in the pathophysiology of hypertrophic remodeling in immature and mature myocardium. Studies in congenital heart disease indicate that oxidative stress appears to be a key feature of hypertrophic remodeling [77], as has also been reported in adults [78], [79]. Mitochondrial dysfunction has also been observed in hypertrophy of the right ventricle associated with congenital heart disease [77], as has likewise been reported in adults [80], [81], [82]. In the following sections, the pathological anatomy and the pathophysiology of immature and mature myocardium in the context of hypertrophic remodeling are discussed. A comparative overview of immature and mature myocardium, with and without hypertrophic remodeling, is provided in Table 1.

**Table 1:** Comparison of healthy and pathological hypertrophic myocardium at immature/maturing and mature stages.

	Immature / maturing myocardium		Mature myocardium	
	*Healthy*	*Pathological Hypertrophy*	*Healthy*	*Pathological Hypertrophy*
*Gross anatomy and function*	In the early postnatal phase remnants from the intrauterine physiology (e.g., comparatively thick right ventricle [192]). During adaptation to postnatal physiology, the gross cardiac structure "normalizes"; for example, mass of the right ventricle decreases after birth [192].	Anatomy varies depending on the underlying cause, for example, biventricular and septal hypertrophy can be found in infants of diabetic mothers [193]. Fixed hypertrophy despite finished adaption to the postnatal circulatory physiology (e.g., persistent hypertrophy of the right ventricle in Tetralogy of Fallot [77] Sudden cardiac death preceding the phenotypic manifestation of myocardial hypertrophy [194]	Wall thicknesses within defined limits, such as an LV wall thickness of <1.5 cm in adults [2], [4]. Normal ventricular dynamics, e.g., no hyperdynamic state at rest. Unremarkable size and shape of the ventricular cavities. No obstruction.	Pathological thickening of the ventricular walls, e.g., left ventricle exceeding 1.5 cm [2], [4]. Hyperdynamic ventricular function [195]. Narrowing of the ventricular chamber even without obstruction [196]. Potentially occurrence of obstruction, e.g., aggravated by a systolic anterior motion of the mitral valve apparatus [47].
*Microscopic features*	The fetal heart is mainly mononucleated, just before birth binucleation begins [197]. Postnatal decrease in right ventricular cardiomyocyte volume [198].	Cardiomyocyte disarray and fibrosis [15]. Non-sarcomeric hypertrophic myocardium is reportedly histologically indistinguishable from sarcomeric HCM [199]. Vacuolization, for example in the context of glycogen storage and glycogen storage-like diseases [200], [201].	Mainly binucleated cardiomyocytes [197]. Highly organized and helical cardiomyocyte architecture [202], [203]. Increasing fibrosis and steatosis during ageing [204].	Cardiomyocyte disarray and fibrosis [15]. Reduction in capillary density and occurrence of vascular dysplasia [101]. Vacuolization [101], [205], [206]. Myofibrillar disorganization [101].
*Cellular and subcellular changes*	Increased ratio of mitochondria to myofibrils [198]. Controlled proliferation [198].	Increased protein kinase B signaling [199], [207], [208]. Variants in α-protein kinase 3 [199], [209], [210]. Desmosomal impairment [199], [209].	Compared with diseased and immature myocardium, mature healthy myocardium exhibits normal cellular and subcellular structure and function, including no signs of energy depletion or mechanoenergetic uncoupling [55], [211], and adequate mitochondrial respiration [212].^#^	Mitochondrial stress and increased ROS [103]. Disrupted creatine kinase system [103]. Altered SRX-DRX equilibrium [51], [213].

The table summarizes and compares features of immature and mature myocardium with and without hypertrophic remodeling. Please note that because of the high specialization of research in the different domains (myocardial metabolism, cardiac ultrastructure, filament mechanics etc.) this summary cannot be exhaustive. Additionally, studies as detailed and deep as for mature cardiomyocytes are in many occasions not (yet) available for immature and maturing cardiomyocytes. **Annotation:** (#) As the non-hypertrophic, not obviously diseased, mature myocardium serves as “control” or “reference” in several studies, for example as so-called “nonfailing donors” [59], studies explicitly outlining and studying in detail the healthy baseline are rare in the given context. Thus, the given references describe the different pathological changes known from mature HCM. **Abbreviations in alphabetical order:** DRX – disordered-relaxed state, HCM – hypertrophic cardiomyopathy, ROS – reactive oxygen species.

### 3.1 Pathological anatomy

As in mature myocardium [4], there are several causes of hypertrophy in immature myocardium [83], [84]. Examples include increased afterload (mature: [85]; immature: [86]) and genetic alterations (mature: sarcomere-associated genes [36]; immature: *RAF1*-mutations causing the Noonan syndrome [87]). In the fetal setting, maternal factors can also cause hypertrophic remodeling, such as maternal diabetes [88]. In addition, intrauterine conditions must be considered in the setting of immature myocardium. For example, an association between intrauterine growth restriction and lifelong cardiovascular morbidity has been observed [89]. Notably, pregnancy may also be associated with physiological hypertrophy of the maternal mature myocardium due to a temporary increase in hemodynamic workload [90], [91]. In both, mature and immature hearts, the hypertrophy predominantly affects the interventricular septum [18], [92], demonstrated in pathological [15], clinical [84], [88], [93], [94], [95], [96] and experimental [97] studies. The importance of involvement of the interventricular septum is underpinned by the fact that septal thickness is considered a baseline echocardiographic parameter [84] and can be predictive of later HCM development [88]. However, the right ventricle can also be involved. For example, immature hypertrophy of the right ventricle can be observed in neonates with congenital hyperinsulinism [98], and adult patients diagnosed with HCM can present with right ventricular outflow tract obstruction [99], [100]. Myocardial hypertrophy can also occur in neonates treated with steroids, as well as in lysosomal storage diseases (e.g., Pompe disease) and other metabolic and neuromuscular diseases, all of which must be taken into account. In general, non-sarcomeric causes of HCM are more common in infants than in older patients. Regarding microscopic structure, immature hypertrophic myocardium shows features comparable to those observed in mature hypertrophic myocardium. Similar to adults, immature hypertrophic myocardium presents with a structurally and functionally altered filament apparatus [87]. In addition, thickened immature myocardium exhibits cardiomyocyte hypertrophy [97] and disarray [15], both well-known of mature HCM [101].

### 3.2 Pathophysiology

In addition to the similarities between immature and mature myocardium described above, there are also important differences, as summarized by Seok and Oh in 2021. For example, at the ultrastructural level, immature cardiomyocytes exhibit fewer sarcomeres and mitochondria, as well as different isoforms of contractile proteins, compared with mature myocardium. These features, together with their capacity for differentiation and proliferation, may make immature cardiomyocytes more susceptible to stimuli such as load [75]. Therefore, at the histopathological and pathoanatomical levels, immature and mature myocardium show both similarities and marked differences too. These differences may lead to distinct pathophysiological behavior of immature myocardium that is clinically relevant. One example is oxygen saturation, which is a major driver of cardiomyocyte maturation, such that chronic hypoxemia results in delayed myocardial maturation. Another example is the capacity of immature myocardium for rapid LV hypertrophy within days to weeks, particularly in response to hemodynamic changes and increased ventricular afterload, potentially aggravated by hypoxia in cases of pulmonary pathologies [75]. Complementing the description of general pathophysiological aspects of immature and maturing myocardium, the following paragraphs discuss pathophysiological hallmarks of HCM in the context of immature and mature myocardium.

#### 3.2.1 Hypercontractility and mechanotransduction

On one hand, the term "hypercontractility" describes a functional state of the cardiomyocytes or of the myocardium more broadly, characterized by increased contraction and ejection. This can be observed even before the manifestation of the overt HCM phenotype [3], [4]. On the other hand, at the subcellular level, analyses of human induced pluripotent stem cell (iPSC)-derived cardiomyocytes carrying the *MYH7*-G256E variant have showed that hypercontractility leads to increased mitochondrial respiration and increased expression of mitochondrial genes [102]. Additionally, extensive analyses of human and murine tissues demonstrated that hypercontractility, mitochondrial reactive oxygen species (ROS) and altered function of the creatine kinase are associated with each other and drive energetic alterations of cardiomyocytes [103]. Furthermore, mechanical forces interfere with processes within the cell in general. Hereby, the nucleus – the stiffest and largest organelle – is connected with the cytoskeleton by a specialized linker complex. Via this link, the nucleus becomes part in several mechanotransduction pathways. One example is the ability of nuclear pore complexes to bind both chromatin and cytoskeletal components, thereby influencing active transcription sites [104]. Also, studies in human iPSC-derived cardiomyocytes have shown that through sarcomere-nucleus mechanotransduction, the contractile state of the sarcomere (whether hyper- or hypocontractile) affects nuclear stiffness via altered expression of lamin A/C, a nucleoskeleton protein [105]. Consistent with these mechanotransduction features associated with hypercontractility, early findings in hypercontractile human iPSC-derived cardiomyocytes with different *MYH7* mutations are published that show activation of the Hippo-YAP signaling [106]. YAP signaling has been demonstrated to lead to hypertrophy via secretion of the cellular communication network factor 2 (CCN2) and uptake of transforming growth factor β (TGFβ) [107] whereas Hippo signaling is reportedly one major signaling pathway during cardiac development [108]. The interaction of hypercontractility with these cascades may be both a potential starting point of the fetal-like reprogramming in hypertrophic mature myocardium and a molecular starting point of hypertrophy during cardiac maturation. Taken together, hypercontractility not only is a (dys)functional state of the myocardium but also is associated with several alterations at the cellular and subcellular levels that finally result in prohypertrophic signaling and hypertrophic remodeling. Given these pathophysiological links, it is plausible that hypercontractility is not only a typical finding in pediatric HCM [109] but may also interfere with the intrauterine cardiac development.

Sarcomere–nucleus mechanotransduction is not the only relevant process in this context; mechanotransduction more broadly is also important. An important molecule in this context is the mechanosensitive PIEZO1 channel. PIEZO1 is found in several key players of hypertrophic remodeling including cardiac fibroblasts, vascular endothelial cells, and cardiomyocytes. Activation of PIEZO1 in cardiomyocytes because of pressure overload (e.g., LV outflow tract obstruction) increases the Ca^2+^ concentration. This, in turn, can activate the transient receptor potential cation channel subfamily melastatin member 4 (TRPM4) which via several steps can cause Ca^2+^/calmodulin-dependent protein kinase II (CAMKII) associated hypertrophic remodeling [110], [111], [112]. Additionally, PIEZO1 activation in cardiac fibroblasts was associated with secretion of the profibrotic and prohypertrophic interleukin-6 [111]. Additionally such an activation of cardiac fibroblasts was found in association with fibroblast trans-differentiation into cardiomyocytes [113]. Thus, mechanotransduction in general is linked with a fetal-like reprogramming.

Importantly, several of these subcellular alterations can be rescued by the application of cardiac myosin-modulating drugs. For example, cardiac myosin inhibition was able to improve cardiomyocyte energy balance [103] and restore nuclear stiffness [105]. This could be a hint that cardiac myosin inhibition in young patients – maybe even in those before phenotypic manifestation of the HCM – could slow or potentially even reduce the extent of manifest myocardial hypertrophy in HCM.

#### 3.2.2 Perturbed metabolism

Metabolic impairment has been identified as a central pathophysiological hallmark of mature HCM [53]. It has also been shown that fetal genes can serve as biomarkers of myocardial hypertrophy in metabolic diseases such as diabetes [27], possibly because of re-establishment of a fetal metabolic gene profile in response to heart failure [28]. This may be particularly relevant considering the massive myocardial workload imposed by adaptive processes in obstructive HCM [51]. Such metabolic parallels between mature and immature myocardium may originate during intrauterine development. This assumption is supported by the association between maternal diabetes and neonatal myocardial hypertrophy [83], [88], [93]. This, in turn, derives from the role of insulin as an embryological growth factor [114]. The importance of insulin in the cardiac development is demonstrated by the fact that high insulin levels can impair the functional capacity of the developing heart [115]. Moreover, observations in the rare Donohue syndrome, also known as leprechaunism, indicate that insulin signaling is important for cardiac development: children with insulin receptor gene mutations present with heart failure, septal hypertrophy [116] and intrauterine growth restriction [117].

**Fig. 1:**
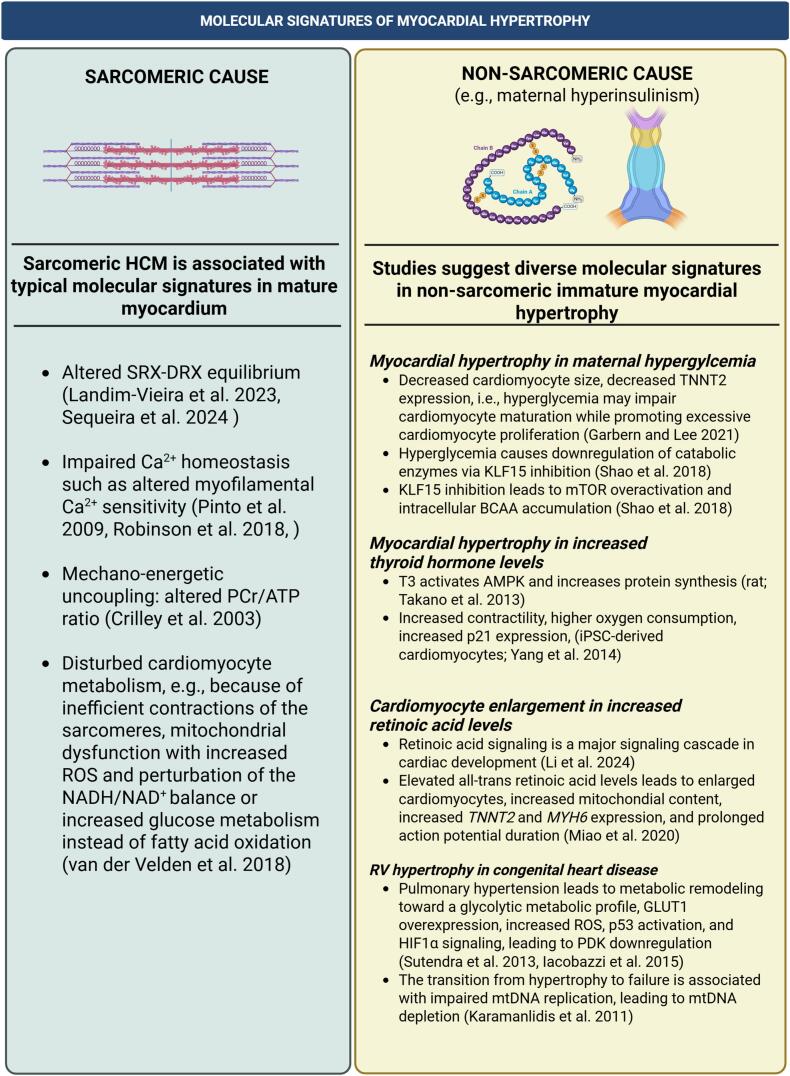
Molecular signatures in sarcomeric and non-sarcomeric myocardial hypertrophy. The figure summarizes and compares key features of the pathophysiology (molecular signature) of hypertrophic immature myocardium due to a sarcomeric (left panel in petrol) or non-sarcomeric cause (e.g., maternal hyperglycemia; right panel in yellow). The summary points out that sarcomeric causes of myocardial hypertrophy show a rather uniform molecular signature whereas the non-sarcomeric causes are associated with a brought variety of molecular changes. Additionally, the summary points out that besides thyroid hormone and insulin discussed in the text several other pathways and mechanisms can be involved in non-sarcomeric hypertrophy of immature myocardium. A holistic summary of all possible mechanisms exceeds the scope of the present narrative review. More detailed overviews are given by reviews more specific for signaling in immature cardiomyocytes such as Gabern and Lee 2021 [186]. Where applicable the model was specified in which the observation was made. **Abbreviations in alphabetical order:** AMPK – adenosine monophosphate-activated protein kinase, ATP – adenosine triphosphate, BCAA – branched chain amino acids, DRX – disordered-relaxed state, GLUT1 – glucose transporter type 1, HIF1α – hypoxia inducible factor 1-α, iPSC – induced pluripotent stem cell, KLF15 – Krüppel-like factor 15, mtDNA – mitochondrial deoxyribonucleic acid, mTOR – mechanistic target of rapamycin, *MYH6* – myosin heavy chain 6, PCr – phosphocreatine, PDK – pyruvate dehydrogenase kinase, ROS – reactive oxygen species, SRX – super-relaxed state, T3 – triiodothyronine, *TNNT2* – troponin T2. **References used in this figure sorted by publication year:** Crilley et al. 2003 [54], Pinto et al. 2009 [187], Karamanlidis et al. 2011 [188], Sutendra et al. 2013 [189], Takano et al. 2013 [134], Yang et al. 2014 [135], Iacobazzi et al. 2015 [77], Robinson et al. 2018 [52], Shao et al. 2018 [190], van der Velden et al. 2018 [53], Miao et al. 2020 [191], Garbern and Lee 2021 [186], Landim-Vieira et al. 2023 [32], Li et al. 2024 [108], Sequeira et al. 2024 [51]. (Created in BioRender. Federspiel, J. M. (2026) https://BioRender.com/m1tonyp; agreement number: *FU29XCNVIB*). (For interpretation of the references to colour in this figure legend, the reader is referred to the web version of this article.)

Again, in-depth studies assessing energetic and metabolic alterations in immature hypertrophic myocardium are lacking, to the authors' knowledge. However, because immature myocardium undergoes maturation and mature myocardium can undergo fetal-like reprogramming, it seems likely that comparable alterations may be observed. The similarities and differences between immature myocardial hypertrophy caused by non-sarcomeric factors, such as maternal diabetes, and sarcomeric mutations are summarized in Fig. 1.

#### 3.2.3 Electrophysiology and cellular energetics

Predisposition to arrhythmias is a pathophysiological hallmark of HCM routed in molecular alterations such as Ca^2+^-dependent action potential remodeling [118], myofilament Ca^2+^ sensitization [119], and energy deprivation [120] These alterations have a major impact on the management of patients, including implantation of an implantable cardioverter-defibrillator in cases of increased risk for fatal arrhythmic events [2], [4], [121]. Thus, in the present context, it is important to emphasize that the immature heart also undergoes electrophysiological [75] and energetic [122] maturation. This includes expansion of the sarcoplasmatic reticulum, whereas T-tubules are lacking [75], [123], along with increased regulation by sarco/endoplasmatic reticulum Ca^2+^ ATPase (SERCA) [75], [122] during the early perinatal period and changes in the expression of several ion channels and ion-channel genes such as KCNH2 and SCN5A [29].

Ion channel and ion-channel genes are particularly important in this context. For example, a study found that sarcomeric mutations are linked with a complex cellular remodeling involving CAMKII-dependent signaling, leading to changes in Ca^2+^ handling proteins and ion channels. These changes, in turn, lead to an increased late Na^+^ current, among other abnormalities, causing altered electrophysiology and Ca^2+^ dynamics in human ventricular cardiomyocytes from adult patients diagnosed with HCM [124]. Such perturbed electrophysiology can also be observed during early stages of cardiac development in mice carrying a *Mybpc3* mutation [125]. However, studies in a heart failure-context suggest that increased myocardial load, such as LV outflow tract obstruction in HCM, and cardiac injury can lead to fetal-like expression of ion channels. For example, L-Type Ca^2+^ channels are down regulated whereas T-type Ca^2+^ channels and *HCN4* are upregulated [126], [127], [128], [129]. A detailed summary of altered electrophysiology in the context of fetal-like gene reprogramming in general is provided by van der Pol et al. [29].

## 4 Fetal-like reprogramming and protein isoform switch

In summary, the pathophysiology of immature and mature myocardium may have comparable endpoints, such as hypercontractility, whereas the underlying molecular causes may differ because of molecular and ultrastructural differences between immature and mature myocardium. In addition, the reactivation of fetal-like gene programs in the diseased adult heart [27], [28], [29], [30] creates a blurred boundary between immature and mature myocardium at the molecular, functional, and structural levels, as outlined below.

### 4.1 General aspects

During cardiac maturation, the heart undergoes a shift from fetal to adult sarcomeric protein isoforms, a process that supports increased cardiac kinetics and contractile power [108], [130], [131], [132]. In this process, thyroid hormone plays a crucial role in regulating the myocardial MHC phenotype through its nuclear receptors, which function as hormone-dependent transcription factors that bind to thyroid hormone-responsive elements in the respective genes. The central role of thyroid hormone in cardiac maturation is reflected by the observation that all genes of the myocardial MHC multigene family are responsive to thyroid hormone. With regard to the MHC isoform switch during maturation, studies have shown that in the ventricles the absence of thyroid hormone is associated with lack of transcription of the α-MHC gene, whereas the β-MHC gene continues to be transcribed [133]. Consistent with central role in cardiac maturation, increased thyroid hormone levels can be associated with immature myocardial hypertrophy (Fig. 1) [134], [135].

Myocardial maturation is also associated with the loss of energy efficiency and regenerative capacity after injury such as myocardial infarction. Therefore, the adult heart is predisposed to adverse remodeling and eventual heart failure [131], [136]. In contrast, neonatal cardiomyocytes retain an inherent proliferative potential and can mount a regenerative response after myocardial infarction [131], [136]. Although this shift may reduce energetic demand and promote growth, fetal sarcomeric isoforms typically exhibit slower cross-bridge kinetics and reduced mechanical forces [28], [137]. Therefore, what begins as an adaptive response to injury can ultimately compromise contractile performance and contribute to progressive cardiac dysfunction [28], [136], [138], [139].

Isoform switching of sarcomeric proteins can be prompted by metabolic remodeling such as the transition from glycolytic to oxidative metabolism, and can occur through shifts in alternative splicing or differential gene expression [140], [141]. Cardiac stress often disrupts these regulatory processes and promotes re-expression of fetal sarcomeric isoforms in adults [140], [141]. A classic example is the developmental shift in MHC isoforms [131], [138], [141], [142]. In rodents, the ventricular myocardium switches from predominantly expressing fetal β-MHC to the faster adult α-MHC isoform [131], [138], [141], [142]. In humans, however, this pattern is different, with fetal β-MHC isoform remaining predominantly expressed into adulthood and very low levels of α-MHC (<5%) present throughout ventricular maturation [131], [138], [141], [142]. This interspecies difference in MHC composition potentially reflects underlying physiological demands, particularly the markedly higher basal heart rates in rodents compared to humans. Consideration of these species-specific regulatory patterns is important when designing therapeutic strategies aimed at regulating fetal-like gene reprogramming in human heart failure.

At the transcriptional level, fetal-like gene reprogramming in heart failure is largely driven by stress-responsive transcription factors such as nuclear factor of activated T cells (NFAT), GATA family members, heart- and neural crest derivatives-expressed protein 2 (HAND2), and myocyte-specific enhancer factor 2 (MEF2) [132], [143]. For example, calcineurin-NFAT signaling is part of the myocardial response to hypertrophic stimuli such as an increase in intracellular Ca^2+^ or oxidative stress [144], [145]. So, increased intracellular Ca^2+^ leads to calmodulin saturation. Calmodulin saturation again leads to dephosphorylation of NFAT family members. Dephosphorylated NFAT enters the nucleus and interferes alongside other transcription factors with the expression of Ca^2+^-inducible genes. [144] Down-stream such hypertrophic stimuli result in fetal-like reprogramming that involves among others the up-regulation of fetal protein isoforms (e.g., β-MHC) [146], the down-regulation of the respective adult isoforms (e.g., α-MHC) [146], and the up-regulation of natriuretic peptides such as the atrial natriuretic factor [146], [147]. Like calcineurin-NFAT signaling, transcription factors of the MEF2 family are part of the response to extracellular stress and thus of relevance in pathological hypertrophy [148]. In parallel, dysregulation of alternative splicing contributes to the re-expression of fetal splice variants [132], [143]. These molecular isoform changes extend beyond sarcomeric MHC to include fetal isoforms of myosin light chain, troponin I, and titin [137]. Collectively, these changes may alter cross-bridge kinetics, Ca^2+^ sensitivity, and diastolic recoil. In parallel with the sarcomeric remodeling, the failing myocardium shifts toward glycolytic metabolism and undergoes extracellular matrix remodeling, thereby promoting fibrosis and ventricular stiffening [108], [139], [149]. Recent research has demonstrate a link between impaired contractility and fibroblast expansion [150]. Notably, Bretherton et al. 2025, identified cardiac fibroblasts as mechanosensitive rheostats that respond to sarcomere hypercontractility in dilated cardiomyopathy [150], providing a framework by which myocyte dysfunction can drive fibroblast expansion and extracellular matrix deposition. This mechanobiological feedback loop likely contributes to the self-perpetuating nature of heart failure progression.

It is important to point out that fetal-like reprogramming involves not only cardiomyocytes but also fibroblasts, and that this reprogramming can even be observed in partially matured myocardium such as in cases of children affected by dilated cardiomyopathy . For example, a study on pediatric dilated cardiomyopathy with analyses on a single-cell level demonstrated that approximately 8% of the fetal transcriptome was re-activated. In parallel, this transcriptional response of the fibroblasts from dilated cardiomyopathy myocardium was associated with downregulated oxidative metabolism and upregulated muscle differentiation [151]. Furthermore, fetal-like reprograming does not only involve gene-expression changes, but also the epigenome and transcriptome remodeling [152], as exemplified by isoform switching in heart disease. Interestingly, the consequences of fetal-like reprogramming and sarcomeric protein isoform switching may be clinically important beyond cardiac disease itself. For example, immature myocardium has been found to be more resilient against acidosis than adult myocardium [153], [154]. Thus, one might hypothesize that myocardium undergoing fetal-like reprogramming might be more resilient in disease states associated with acidosis such as diabetic keto-acidosis or following resuscitation.

### 4.2 Reprogramming and fetal-like patterns in hypertrophic cardiomyopathy

As early as the 2000s, reviews and editorials pointed out that fetal-like reprogramming in HCM is associated with progression to heart failure and impaired Ca^2+^ cycling at the cellular level [155], [156]. Additionally, in 2015, murine studies in a double-transgenic tetracycline-controlled transactivator x α-MHC^R403Q^ model demonstrated that the early postnatal period is crucial for the development of the HCM phenotype, and that irreversible defects occurring during this period may drive LV hypertrophy [157]. Recently, multi-omics studies by Gao et al. assessing human and murine HCM tissues demonstrate that such reprogramming also takes place in HCM [158]. For example, the group demonstrated that several transcription factors, such as early growth response 1 (EGR1), show fetal-like patterns in their binding motifs. Additionally, they demonstrated that inhibition of these affected transcription factors leads to a less pronounced HCM phenotype alongside reversal of fetal-like reprogramming. Based on these findings, the authors concluded that fetal-like reprogramming may also represent a potential therapeutic target in HCM [158]. In 2023, Liu and colleagues performed extensive single-nucleus ribonucleic acid (RNA)-seq and spatial transcriptomic analyses using tissue of patients diagnosed with HCM and healthy donors [159]. Interestingly, their observations were consistent with findings in pediatric dilated cardiomyopathy [151]: the two studies found alterations in both cardiomyocytes and fibroblasts [151], [159]. In the study on HCM, clustering analysis identified two sub-populations of cardiomyocytes. One of these subpopulations exhibited a re-activated fetal-like gene program, including genes such as *ACTA1*, which encodes for skeletal α-actin. These changes, however, were associated with failing cardiomyocytes [159]. A study by Han et al. from 2025 created a “ratiometric catalog of protein isoforms” using mass spectrometry with a proteome-wide protein ratio quantification. With this advanced technique, they were able to identify a previously unknown isoform usage of mitochondrial proteins and gene-expression-regulating proteins in murine cardiac disease [160].

## 5 General therapeutic aspects of immature and mature hypertrophic myocardium

Translational research in HCM not only addresses mature myocardium but also considers cardiac development. As early as 1977, it was proposed that embryological factors provide the foundation for the development of myocardial hypertrophy and architectural changes in the myocardium, such as cardiomyocyte disarray [15], [101]. This review has outlined similarities between immature and mature myocardium, both healthy and diseased, at the structural level (such as LV thickening) and the functional level (for example, hypercontractility and metabolic alterations). The similarities between immature and mature hypertrophic myocardium support the idea that the frequent hypertrophy of the interventricular septum observed in HCM may be rooted in cardiac embryogenesis.

However, compared with mature hypertrophic myocardium, relatively little is known about the precise molecular and biophysical basis of maladaptive remodeling in immature myocardium in HCM. For example, in adults, it has been shown that different pathways, such as the ERK/MAPK pathway influence whether myocardial remodeling becomes dilated or hypertrophic. Such key regulatory elements have not yet been defined in immature myocardium with the same level of detail as in mature myocardium.

Why, then, does HCM usually manifest in adulthood [3], if embryonic factors are important? Assuming an embryological origin of HCM [15], [18], resulting in early postnatal alterations of the myocardial layer that subsequently promote LV hypertrophy [157], the mechanical stress imposed by isometric septal contraction may, over time, act as a “second hit”. This secondary hit could induce fetal-like reprogramming processes, thereby accelerating a self-perpetuating molecular cascade, such as ERK1/2 signaling as a mediator of concentric remodeling in HCM [161]. Such molecular cascades could further drive the progression of myocardial hypertrophy.

Given its pathophysiological relevance, the fetal-like reprogramming may represent a potential leverage point for the development of targeted pharmacological treatment strategies [159]. The treatment of patients diagnosed with HCM depends on the specific manifestation of the disease, including the degree of LV outflow tract obstruction, LV ejection fraction, occurrence of arrhythmias, and the associated risk of sudden cardiac death. Taking these factors into account, treatment may include medical therapy such as β-blockers or Ca^2+^ antagonists in older children and adults. Notably, verapamil is not recommended in infants because of serious pro-arrhythmic effects in this age group [162], [163]. Treatment may also include device therapy, such as implantation of an implantable cardioverter-defibrillator or cardiac resynchronization therapy, septal reduction therapy, including alcohol septal ablation or surgical septal myectomy or heart transplant. [2] Septal reduction therapy has been demonstrated to be effective treatment in both pediatric [164] and adult [12] patients.

### 5.1 Myosin inhibition therapy in immature and mature myocardium

The mechanism of action of the cardiac myosin inhibition therapy can be traced to the cross-bridge kinetic alterations in HCM. Myosin can adopt one of two energy states: the super-relaxed state (SRX) with low ATPase activity, and the disordered-relaxed state (DRX) with faster ATPase activity. The ratio of these two states is a critical regulator of contraction, and SRX myosin acts as an important reserve for force generation [165]. The SRX/DRX myosin ratio is relevant as myosin inhibitors such as mavacamten or aficamten, increase the proportion of SRX myosin, thereby acting as contractile depressants. These show benefits in patients diagnosed with obstructive HCM but interestingly, have failed to show clinical benefits in non-obstructive HCM [7], [8], [46].

PKA phosphorylation of MyBPC3, particularly at serine 282, has been demonstrated to recruit myosin heads from SRX to DRX, although the reverse is not necessarily true [166]. A reduction in the proportion of SRX myosin was previously thought to be one of the hallmarks of HCM [65], [66], but recent studies in tissue from patients *MYBPC3* missense variants and/or thin-filament variants have shown the opposite [167], [168], [169]. For example, in silico human models of *TNNT2*-R92Q and *TNNI3*-R21C predicted an increase in SRX myosins in HCM [169], whereas experimental data from murine myocardium carrying *TNNT2*-I79N showed the opposite pattern, with a decrease in SRX myosins [32]. This discrepancy may reflect the limitations of current computational models, which do not fully capture disease-related tissue remodeling observed in animal models, as well as the possibility that different variants or residue locations influence the SRX/DRX equilibrium in distinct ways.

Cardiac myosin inhibitors, including mavacamten and aficamten [2], [7], restore the balance between activated and inactive myosin states by increasing the physiological proportion of myosin in a SRX state [170]. Cardiac myosin inhibition is also promising for pediatric patients [171], [172], as supported by experimental data generated using iPSC-derived cardiomyocytes from pediatric patients with HCM [173]. Correspondingly, mavacamten is currently being evaluated for symptomatic adolescent patients diagnosed with obstructive HCM. [174]. Given the molecular and structural similarities between mature and immature myocardium, evidence of therapeutic benefit in these patients would further support the pathophysiological similarities between the two. Looking ahead, the importance of these similarities, as well as the impact of cardiac development on HCM, is further emphasized by clinical case reports describing early steps toward intrauterine treatment of fetuses diagnosed with HCM, for example with the β-blocker propranolol, a medication commonly used in children with obstructive HCM [95].

## 6 Limitations and future perspectives

Research on the fetal-like reprogramming can be traced back to the first observations of the fetal-to-adult class switch from α-MHC to β-MHC in 1972 [132]. Despite more than 40 years of research in the fetal-like gene reprogramming still “remains one of the least understood aspects of cardiac developmental biology” (page 193 [132]) according to a recent major review article by Porrello et al. [132]. For example, to the authors knowledge there is no study available directly comparing the fetal-like reprogramming in different up-stream triggers such as sarcomeric mutations or arterial hypertension. Nevertheless, several signaling pathways and regulatory systems relevant to both hypertrophic remodeling and fetal-like reprogramming have been identified. For example, Davis et al. showed that increased tension on the myofilaments can induce ERK1/2 signaling, leading to HCM remodeling. In addition, WNT/β-catenin signaling, which regulates factors such as MEF2c and HAND2, is central for the regulatory control of the second heart field during cardiac formation, as summarized by Kodo and Yamagishi [25]. Moreover, it must be considered that different signaling cascades are activated at different time points during cardiac development. For example, MEF2C is considered a key regulator in the cardiac crescent, linear heart tube, and looping heart, but not at the stage of cardiac septation and chamber specialization [175]. These pathways also vary by anatomical location. For example, during cardiac mesoderm specification, the splanchnic mesoderm secretes different ligands than the neuroectoderm [175]. Taken together, we currently know a variety of signaling cascades involved in hypertrophic remodeling and fetal-like reprogramming, but we do not yet have a holistic understanding of when, where, and how these cascades interlace and/or diverge.

Table 2 summarizes articles specifically reporting aspects of developmental and pathophysiological signaling, associated mechanisms such as mechanotransduction, and pathological and pathophysiological aspects of cardiac development. To better understand the temporal and spatial changes that occur during development and maturation, longitudinal studies following these processes would be helpful. However, longitudinal assessment of human cardiac maturation is challenging, for example because of ethical and legal challenges and restrictions surrounding the scientific use of embryos and embryo-like structures [176], [177]. Thus, as detailed throughout the manuscript, observations made in various models and study designs are reported in the present narrative review, including different sarcomeric mutations, rodent species, and human iPSC-derived cardiomyocytes. Correspondingly, in a systematic review of HCM models published from 1977 to 2023, van den Dolder et al. identified approximately 630 different models. Most of these models were mouse models (*n* = 304). They observed an increasing number of cell-based models from 2010 onward. However, they found that, in both cell-based and animal models, most studies do not report all disease hallmarks, such as cardiac and cardiomyocyte hypertrophy, fibrosis or impaired relaxation. They additionally pointed out that, by design, cell-based models such as human iPSC-derived cardiomyocytes cannot examine changes at the organ level. For example, they can assess cardiomyocyte hypertrophy but not cardiac hypertrophy [178]. Thus, no currently available model allows longitudinal assessment of all molecular, structural, and functional aspects of cardiac development and maturation, with and without sarcomeric mutations, while using a reasonable number of animals or cells. Most of the mechanistic data outlined in the present narrative review derive from several models, especially rodent models. Therefore, it must be emphasized that there are differences between the human and rodent heart. For example, with few exceptions, such as the tips of the papillary muscles, the murine heart predominantly expresses α-MHC [179], whereas the human ventricular myocardium predominantly expresses β-MHC [180]. Because of these differences, further research is needed to determine how murine mechanisms translate to human myocardium. A promising approach in this context is the use of human iPSC-derived cardiomyocytes. By design, this in vitro model promotes and requires differentiation of pluripotent stem cells toward cardiomyocytes, and therefore cardiomyocyte maturation [181]. Additionally, human iPSC-derived cardiomyocytes are (still) an active area of current research, leading to fast evolvement and improvement of these models [181], [182]. Furthermore, such models allow investigation of aspects central for hypertrophic remodeling in maturing myocardium (e.g., mechanotransduction [183], Ca^2+^ handling [184], and interaction with cardiac fibroblasts [185]). Although iPSC-derived models – in their current state – cannot fully mimic maturation and hypertrophic remodeling at the organ level, they appear to be key tools for understanding the molecular basis of hypertrophic remodeling in immature and maturing cells caused by sarcomeric and non-sarcomeric mechanisms – especially considering the differences of human and other mammalian hearts.

**Table 2:** Summary of articles in the context of signaling in cardiac development, maturation and disease.

Article	Covered aspects
Yamagishi et al. (2009) [23]	Summary of the pathoanatomic connection of cardiac development and congenital heart disease with a focus on Tbx1 (i.e., transcription factor, member of the T-box family) and its regulation in the context of congenital heart disease.
Kodo and Yamagishi (2011) [25]	Summary of basic morphological aspects such as distribution and localization of the primary and secondary heart field within the embryo and the heart tube. Additionally, the article covers a summary of the regulatory network of the secondary heart field with Wnt/β-catenin as one component.
Di Salvo and Haldar (2014) [143]	Detailed and pragmatic summary of how epigenetics work (e.g., differentiation between “writers” and “erasers” etc.) and its implication for heart failure in general.
Paige et al. (2015) [175]	Brief summary of regulating factors and regulating networks involved in the differentiation of cardiomyocytes at different time points (e.g., cardiac crescent, linear heart tube etc.) and different sites (e.g., neuroectoderm, splanchnic mesoderm etc.) including a “family tree” of the cardiomyocytes' lineage diversification
van der Velden et al. (2018) [53]	Brought and detailed summary of metabolic alterations in HCM.
Janota, Calero-Cuenca and Gomes (2020) [104]	Detailed summary and description of the nucleus in mechanotransduction in general including explanations how the nucleus is linked with the contractile apparatus of the cell and the role of the nuclear pore complex in cellular mechanotransduction.
van der Pol et al. (2020) [29]	Review on the fetal-like reprogramming in heart failure covering several topics such as metabolism and electrophysiology including the expression of ion channels.
Garbern and Lee (2021) [186]	Brought review on the metabolism in cardiomyocyte maturation with a brought outline of associated signaling (thyroid hormones, retinoic acid etc.)
Seok and Oh (2021) [75]	Brought outline of physiological and pathophysiological aspects of cardiomyocyte maturation focusing on HCM in infants.
Hong and Zhang (2022) [214]	Detailed review on various transcriptions factors in cardiac remodeling in general. They also summarize which transcription factors can be associated with which process (e.g., NFAT has been found in associated with the expression of fetal genes and fibrosis) and which genes.
Coste and Delmas (2024) [111]	Detailed review on the mechanosensitive ion channels PIEZO1 and PIEZO2 including general aspects of mechanotransduction including cardiac fibroblasts and vascular endothelial cells.
Ewoldt et al. (2024) [181]	Discussion of the current challenges in research using human iPSC-derived cardiomyocytes.
Khalilimeybodi, Saucerman, and Rangamani (2024) [215]	Extensive and detailed modeling approach to understand the regulatory networks involved in the hypertrophic remodeling in HCM.
Li et al. (2024) [108]	Detailed summary on the molecular mechanisms of diseases related to cardiac development including an outline of historic milestones, a summary of which models were used to make advances in this field, and a detailed summary on the signaling cascades involved in different states of cardiac development.
Sequeira et al. (2024 [51]	Discusses pathophysiological aspects of a perturbed DRX-SRX equilibrium in HCM.
Porrello et al. (2026) [132]	To the authors' knowledge the most recent summary and discussion of the fetal-like gene reprogramming in general and without particular focus on HCM including a comparison between mature and immature cardiomyocytes and the role of inflammation in the context of fetal-like reprogramming.

The articles are sorted by publication year. The summary of covered aspects focuses on the context of signaling in the context of cardiac development, myocardial maturation, hypertrophic remodeling, and fetal-reprogramming. **Abbreviations in alphabetical order:** DRX – disordered relaxed state, HCM – hypertrophic cardiomyopathy, iPSC – induced pluripotent stem cells, NFAT – nuclear factor of activated T-cells, SRX – super-relaxed state, Wnt – wingless-related integration site.

## 7 Summary and conclusion

The literature reviewed indicates that immature and mature myocardium affected by sarcomeric variants differ at the molecular level, including SERCA regulation and T-tubule formation [75]. However, murine studies suggest that the early postnatal period is a critical window during which sarcomeric alterations emerge and may later drive LV hypertrophy [157]. Given the shared down-stream structural and functional abnormalities observed in HCM, including cardiomyocyte disarray [15] and hypercontractility in both pediatric [109] and adult patients [2], fetal-like reprogramming may represent a molecular convergence point linking immature and mature myocardium in the progression toward maladaptive hypertrophic remodeling. Accordingly, further research into embryonic factors, such as early intrauterine and postnatal sarcomeric alterations and fetal-like reprogramming, may provide new opportunities for HCM-specific targeted therapies.

## CRediT authorship contribution statement

**Jan M. Federspiel:** Writing – review & editing, Writing – original draft, Visualization, Conceptualization. **Steven Lee Medarev:** Writing – review & editing, Writing – original draft, Investigation. **Jochen Pfeifer:** Writing – review & editing, Writing – original draft, Investigation. **Vivek P. Jani:** Writing – review & editing, Writing – original draft, Investigation. **José Renato Pinto:** Writing – review & editing, Writing – original draft, Investigation. **Maicon Landim-Vieira:** Writing – review & editing, Writing – original draft, Validation.

## Funding

This work was supported by the 10.13039/100000050National Heart, Lung, and Blood Institute HL160966 to J.R.P.

## Declaration of competing interest

The authors declare the following financial interests/personal relationships which may be considered as potential competing interests: Pinto reports financial support was provided by National Heart, Lung, and Blood Institute (HL160966). If there are other authors, they declare that they have no known competing financial interests or personal relationships that could have appeared to influence the work reported in this paper.

## Data Availability

The present manuscript does not contain original data. The literature building the basis of the present narrative review is cited throughout the text.
